# Monthly Abortions

**Published:** 1876-08

**Authors:** 


Monthly Abortions.—Dr. Mary Putnam-Jacobi presented a small specimen to the New York Pathological Society which had been discharged from the uterus of a married lady during one of her monthly periods. As similar masses, partly resembling clots and partly resembling membranes, had been voided every month, and as the patient was always regular in her catamenia, she was supposed to be suffering from simple dysmenorrhœa. The passage of the clot, as is usual on such occasions, was always accompanied with pain. The microscopic examination of the specimen in question showed the existence of flat epithelial cells imbeded in a very fine matrix, but none of the cylindrical cells of the uterine cavity were found. The clot was then carefully washed, when the appearance of a very minute blood-stained embryo was seen, with head, neck, extremities, and umbilical cord. The doctor believed that this product of conception had been destroyed by hemorrhage as soon as formed, and thought it by no means impossible that the previous attacks of dysmenorrhœa could be explained in a like manner; in other words, that the patient had been in all probability suffering from monthly abortions.—*Med. Record.*

